# Cardiovascular events and all-cause mortality in a cohort of 57,946 patients with type 2 diabetes: associations with renal function and cardiovascular risk factors

**DOI:** 10.1186/s12933-015-0204-5

**Published:** 2015-04-18

**Authors:** Lucia Cea Soriano, Saga Johansson, Bergur Stefansson, Luis A García Rodríguez

**Affiliations:** Spanish Centre for Pharmacoepidemiologic Research (CEIFE), Almirante 28-2, E 28004 Madrid, Spain; AstraZeneca R&D, Mölndal, Sweden

**Keywords:** Chronic kidney disease, Estimated glomerular filtration rate, Ischemic stroke, Mortality, Myocardial infarction, Type 2 diabetes

## Abstract

**Background:**

Diabetes and chronic kidney disease (CKD) are independent predictors of death and cardiovascular events and their concomitant prevalence has increased in recent years. The aim of this study was to characterize the effect of the estimated glomerular filtration rate (eGFR) and other factors on the risk of death and cardiovascular events in patients with type 2 diabetes.

**Methods:**

A cohort of 57,946 patients with type 2 diabetes who were aged 20–89 years in 2000–2005 was identified from The Health Improvement Network, a UK primary care database. Incidence rates of death, myocardial infarction (MI), and ischemic stroke or transient ischemic attack (IS/TIA) were calculated overall and by eGFR category at baseline. eGFR was calculated using the Modification of Diet in Renal Disease (MDRD) study equation. Death, MI and IS/TIA cases were detected using an automatic computer search and IS/TIA cases were further ascertained by manual review of medical records. Hazard ratios (HRs) and their corresponding 95% confidence intervals (CIs) for death, MI, and IS/TIA associated with eGFR category and other factors were estimated using Cox regression models adjusted for potential confounders.

**Results:**

Overall incidence rates of death (mean follow-up time of 6.76 years), MI (6.64 years) and IS/TIA (6.56 years) were 43.65, 9.26 and 10.39 cases per 1000 person-years, respectively. A low eGFR (15–29 mL/min) was associated with an increased risk of death (HR: 2.79; 95% CI: 2.57–3.03), MI (HR: 2.33; 95% CI: 1.89–2.87) and IS/TIA (HR: 1.77; 95% CI: 1.43–2.18) relative to eGFR ≥ 60 mL/min. Other predictors of death, MI and IS/TIA included age, longer duration of diabetes, poor control of diabetes, hyperlipidemia, smoking and a history of cardiovascular events.

**Conclusions:**

In patients with type 2 diabetes, management of cardiovascular risk factors and careful monitoring of eGFR may represent opportunities to reduce the risks of death, MI and IS/TIA.

**Electronic supplementary material:**

The online version of this article (doi:10.1186/s12933-015-0204-5) contains supplementary material, which is available to authorized users.

## Background

Diabetes and chronic kidney disease (CKD) are independent predictors of death and cardiovascular events [1-3]. The prevalence of CKD in individuals with diabetes has increased in recent years and studies have estimated that about 25–30% of patients with type 2 diabetes have CKD stages 3–5 in the UK [4,5]. Additionally, type 2 diabetes is the most common reason for renal replacement therapy in the Western world [6].

The potential association between impaired renal function (as measured by the estimated glomerular filtration rate [eGFR]) and all-cause mortality and/or incidence of cardiovascular events has been thoroughly studied in the general population [1,7-11], in patients with cardiovascular diseases [12-16] and in those with impaired renal function [17,18]. Although the association between decreased renal function and death in individuals with type 2 diabetes has been studied to some extent [19-24], data on cardiovascular mortality and morbidity remain scarce in this patient population [19,20,24-28].

The aim of this study was to determine the incidences of death, myocardial infarction (MI), and ischemic stroke or transient ischemic attack (IS/TIA) in a population of individuals with prevalent type 2 diabetes, overall and according to eGFR calculated from baseline measurement of creatinine. Risks of death, MI and IS/TIA adjusted for potential confounders (including cardiovascular risk factors) and associated with eGFR baseline measurement was also estimated. Other predictors of death and cardiovascular outcomes were also identified overall and for each CKD stage.

## Methods

### Data source

A retrospective cohort study was performed using data from The Health Improvement Network (THIN), a computerized primary care database containing anonymized records for individuals currently registered with participating primary care practices in the UK. THIN is age, sex and geographically representative of the UK population [29] and has been extensively validated for epidemiological studies [30,31]. Anonymized data on patients are systematically recorded by participating primary care physicians (PCPs) as part of their routine patient care and regularly delivered to THIN for use in research projects. The computerized information includes demographics, details of PCP visits, diagnoses, referrals to specialists and hospital admissions, and a free-text section. Participating practices are required to record prescriptions and new courses of therapy. THIN also provides a standardized system for the reliable and comprehensive recording of additional health data such as results of laboratory tests (including serum creatinine concentration, when appropriate). The Read classification is used to code specific diagnoses [32], and a drug dictionary based on data from the Multilex classification is used to record prescriptions [33]. The collection of data in THIN database was approved by a Multicentre Research Ethics Committee in the UK (MREC reference number: 08/H0305/49).

### Study design

A cohort of patients with diagnosed type 2 diabetes who were aged 20–89 years between January 1, 2000 and December 31, 2005 was identified from THIN (n = 64,755). The wide age range was chosen to include the general adult population with prevalent type 2 diabetes. Eligible individuals were required to be registered for at least 3 years with their PCP, to have had at least one visit recorded in the past 3 years, and to have a recorded prescription history of 3 years or more. Patients were included in the study cohort if they had at least one creatinine measurement of 10–250 μmol/L recorded between 1 January 2000 and 31 December 2005. Patients with a record of hemodialysis (n = 109) or renal transplant (n = 60) before their start date were excluded, and patients with a recorded incidence of hemodialysis or renal transplant during follow-up were censored from the analysis (n = 108 for hemodialysis and n = 5 for renal transplant).

Among all individuals with type 2 diabetes meeting these criteria (n = 57,957), 56,693 (97.8%) had a first recorded creatinine measurement of 10–250 μmol/L. The date of this first recorded creatinine measurement was defined as their start date. The remaining 1264 individuals (2.2%) had a first creatinine measurement < 10 μmol/L (n = 1161) or > 250 μmol/L (n = 103), and a subsequent measurement within the range 10–250 μmol/L. The date of their first serum creatinine measurement between 10 and 250 μmol/L was defined as their start date. The mean and median times from their first recorded measurement to their start date were 341 days and 202 days, respectively. All patients were followed up from their start date to the first occurrence of either of the following endpoints in three different analyses based on the studied outcome: outcome of interest (death, MI or IS/TIA), reaching the age of 90 years, or end of the study period (December 31, 2010). It should be noted that 11 patients were excluded from the final cohort (seven individuals who had died at start date, and four who had no visits during follow-up), resulting in a final cohort of 57,946 patients.

### Ascertainment and duration of type 2 diabetes

Type 2 diabetes diagnosis was based on the Read classification codes assigned by the PCP or use of hypoglycemic drugs or insulin. For the majority of cases, the type of diabetes was specifically reported by the physician. If the physician used an unspecific diagnostic code (e.g., diabetes mellitus), we reviewed the patient’s medical record back to one year before the diagnosis including any referral letters and physicians’ free-text comments to assign the type of diabetes. If the age of onset was ≤ 35 years and the patient had one or more prescriptions for insulin and less than one year of oral hypoglycemic treatment, the case was classified as type 1 diabetes. Conversely, if the age of onset was ≥ 50 years and the patient used oral hypoglycemic treatment for at least 1 year, the case was classified as type 2 diabetes. A previous THIN study with a similar diabetes ascertainment algorithm estimated a diabetes prevalence that closely matched the prevalence in the Health Survey of England, which is a national population survey [34,35].

Duration of diabetes was defined as the time interval between the first ever recorded entry for type 2 diabetes in the database (including treatment for diabetes) and the start date (date of the first ever valid recorded serum creatinine measurement). Duration of diabetes was categorized into five groups: < 1 year, 1–4 years, 5–9 years, 10–14 years and ≥ 15 years.

### Estimated glomerular filtration rate

The modification of diet in renal disease (MDRD) study formula and the Cockcroft–Gault formula are routinely used to calculate eGFR from serum creatinine concentration. In this study, the eGFR at baseline was calculated using the MDRD study formula (eGFR = 186 × Cr^–1.154^ × age^–0.203^ × 1.212 [if black] × 0.742 [if female], where Cr is the serum creatinine concentration in mg/dL). Ethnicity is not recorded in THIN, hence the same formula was used for all patients (eGFR = 186 × Cr^–1.154^ × age^–0.203^ × 0.742 [if female]) to classify them into five subgroups according to their baseline eGFR: < 15 mL/min (CDK stage 5), 15–29 mL/min (CKD stage 4), 30–44 mL/min (CKD stage 3B), 45–59 mL/min (CKD stage 3A) and ≥ 60 mL/min (CKD stages 1 and 2, or no CKD).

### Myocardial infarction ascertainment

An automatic computer search for specific Read codes was used for the ascertainment of MI cases. Previous studies using this method have shown a very high specificity for MI, resulting in a confirmation rate greater than 90% when validated with the PCP via a questionnaire [36]. Therefore, additional steps of validation of the ascertainment of MI cases, such as manual review of patients’ profiles or validation with a questionnaire, were not carried out in the present study. A total of 3435 cases of MI were identified.

### Ischemic stroke ascertainment

The predictive value of computer-detected IS/TIA is lower than that for other outcomes such as MI owing to the level of misclassification of diagnoses using Read codes. Therefore, we used a multistep approach to ascertain IS/TIA cases (see Additional file 1 for a detailed description). Briefly, a computer search using Read codes suggestive of IS/TIA identified 4799 potential cases. Among these cases, 902 were matched to patients reviewed in other projects in which we looked at a diagnosis of IS/TIA in THIN [37,38]; 653 were classified as non-cases and 249 as cases. For the remaining 3897 patients, the cases of IS/TIA were ascertained in a stepwise fashion by first searching for indicators of hospitalization or referral and then searching for indicators of symptoms, diagnostic procedures and new treatment related to stroke in the 30 days before and after the date of the computer-detected IS/TIA. Finally, the profiles (including free text) of sample patients from different subgroups were manually reviewed to validate the ascertainment of cases. Overall, we identified 3785 cases of IS/TIA.

### Data collection

Data on demographic variables including sex, age, smoking status, alcohol use, body mass index (BMI) and Townsend deprivation index (a measure of material deprivation within a population that takes into account four main variables: unemployment rate, car ownership, home ownership and household overcrowding) [39] were collected any time before the start date. Exposure to drugs was collected before the start date and categorized as follows: current use, when the supply of the most recent prescription lasted until the start date or ended in the 90 days before the start date; recent use, when supply of the most recent prescription ended more than 90 days before the start date; and non-use, when there was no recorded use any time before the start date. Data on healthcare service use (PCP visits, referrals and hospitalizations) were collected for the year before the start date. Information on comorbidities was collected any time before the start date. Data on levels of glycated hemoglobin (HbA_1c_) were collected for the year before the start date. Patients were classified into subgroups according to the HbA_1c_ data recorded closest to their start date: < 7.00%, 7.00–7.99%, 8.00–8.99%, 9.00–9.99%, 10.00–10.99% and ≥ 11.00%. Individuals without a recorded level of HbA_1c_ in the year before their start date were included in the ‘missing’ category.

### Statistical analysis

Incidence rates of death, MI and IS/TIA were calculated overall and by eGFR categories. Kaplan–Meier survival curves for all-cause mortality, MI and IS/TIA were calculated overall and according to eGFR category. Hazard ratios (HRs) and their 95% confidence intervals (CIs) were calculated using Cox proportional hazard models adjusted for sex, age, BMI, smoking status, hyperlipidemia, hypertension, history of MI, history of IS/TIA, history of ischemic heart disease (excluding MI), eGFR category, duration of diabetes, HbA_1c_ category, and polypharmacy (in the month before the start date). A two-sided *p* value < 0.05 was considered to be statistically significant. Statistical analyses were performed using the Stata package version 12.0 (StataCorp LP, College Station, TX, USA).

## Results

### Baseline characteristics and comorbidities

Table 1 shows the main baseline characteristics of patients with type 2 diabetes included in this study, according to their eGFR category. Almost 70% of patients had an eGFR ≥ 60 mL/min and about 9% had an eGFR of 15–44 mL/min (CKD stages 3B and 4). Overall, the mean age at start date was 65.7 years and there were more men than women in the study cohort (55.4% and 44.6%, respectively). However, there were more women than men in the subgroup of patients with an eGFR < 60 mL/min (58.7% and 41.3%, respectively). Over 75% of patients were overweight or obese (BMI ≥ 25 kg/m^2^) and over 65% were using 2–9 drugs in the month before their start date. About 70% of patients had had diabetes for 1–9 years at their start date, whereas about 5% had had diabetes for < 1 year. Among patients with a record of HbA_1c_ level, about 60% (29,476/48,858) had an HbA_1c_ level ≥ 7%.

**Table 1:** **Baseline characteristics, overall and according to estimated glomerular filtration rate category**

			**eGFR category (mL/min)**							
	**Overall (N = 57,946)**		**15–29 (n = 972)**		**30–44 (n = 4326)**		**45–59 (n = 12,614)**		**≥ 60 (n = 40,034)**	
	**Number**	**%**	**Number**	**%**	**Number**	**%**	**Number**	**%**	**Number**	**%**
Sex										
Men	32,117	55.4	315	32.4	1654	38.2	5434	43.1	24,714	61.7
Women	25,829	44.6	657	67.6	2672	61.8	7180	56.9	15,320	38.3
Age at start date (years)										
20–39	1269	2.2	0	0.0	3	0.1	30	0.2	1236	3.1
40–49	4454	7.7	8	0.8	27	0.6	187	1.5	4232	10.6
50–59	10,729	18.5	37	3.8	175	4.0	1067	8.5	9450	23.6
60–69	17,889	30.9	196	20.2	883	20.4	3623	28.7	13,187	32.9
70–79	16,933	29.2	378	38.9	1882	43.5	5303	42.0	9370	23.4
80–89	6672	11.5	353	36.3	1356	31.3	2404	19.1	2559	6.4
Smoking status										
Non-smoker	30,175	52.1	561	57.7	2441	56.4	7028	55.7	20,145	50.3
Current	10,128	17.5	104	10.7	533	12.3	1671	13.2	7820	19.5
Former	15,011	25.9	240	24.7	1105	25.5	3367	26.7	10,299	25.7
Unknown	2632	4.5	67	6.9	247	5.7	548	4.3	1770	4.4
BMI (kg/m^2^)										
15–19	915	1.6	18	1.9	84	1.9	234	1.9	579	1.4
20–24	9546	16.5	174	17.9	818	18.9	2223	17.6	6331	15.8
25–29	21,011	36.3	297	30.6	1521	35.2	4757	37.7	14,436	36.1
≥ 30	22,959	39.6	362	37.2	1487	34.4	4529	35.9	16,581	41.4
Unknown	3515	6.1	121	12.4	416	9.6	871	6.9	2,107	5.3
Number of drugs										
≤1	15,007	25.9	107	11.0	626	14.5	2708	21.5	11,566	28.9
2–4	20,458	35.3	224	23.0	1185	27.4	4121	32.7	14,928	37.3
5–9	18,680	32.2	461	47.4	1920	44.4	4748	37.6	11,551	28.9
10–14	3335	5.8	158	16.3	513	11.9	906	7.2	1758	4.4
≥15	466	0.8	22	2.3	82	1.9	131	1.0	231	0.6
Duration of diabetes (years)										
<1	2848	4.9	26	2.7	138	3.2	557	4.4	2127	5.3
1–4	23,201	40.0	208	21.4	1311	30.3	4545	36.0	17,137	42.8
5–9	17,123	29.5	298	30.7	1271	29.4	3786	30.0	11,768	29.4
10–14	8503	14.7	184	18.9	824	19.0	2095	16.6	5400	13.5
≥15	6271	10.8	256	26.3	782	18.1	1631	12.9	3602	9.0
HbA_1c_ (%)										
<7.00	19,382	33.4	272	28.0	1463	33.8	4480	35.5	13,167	32.9
7.00–7.99	12,823	22.1	203	20.9	964	22.3	2922	23.2	8734	21.8
8.00–8.99	7221	12.5	111	11.4	480	11.1	1565	12.4	5065	12.7
9.00–9.99	4634	8.0	66	6.8	302	7.0	877	7.0	3389	8.5
10.00–10.99	2497	4.3	44	4.5	142	3.3	433	3.4	1878	4.7
≥11	2301	4.0	43	4.4	134	3.1	409	3.2	1715	4.3
Missing	9088	15.7	233	24.0	841	19.4	1928	15.3	6086	15.2
PCP visits										
0–4	8018	13.8	87	9.0	471	10.9	1508	12.0	5952	14.9
5–9	17,371	30.0	229	23.6	1120	25.9	3640	28.9	12,382	30.9
10–19	23,024	39.7	355	36.5	1717	39.7	5228	41.4	15,724	39.3
≥20	9533	16.5	301	31.0	1018	23.5	2238	17.7	5976	14.9
Referrals										
0–4	50,987	88.0	750	77.2	3634	84.0	10,976	87.0	35,627	89.0
5–9	5525	9.5	162	16.7	513	11.9	1287	10.2	3563	8.9
10–19	1315	2.3	55	5.7	162	3.7	325	2.6	773	1.9
≥20	119	0.2	5	0.5	17	0.4	26	0.2	71	0.2
Hospitalizations										
None	52,092	89.9	735	75.6	3676	85.0	11,206	88.8	36,475	91.1
1–2	5013	8.7	176	18.1	524	12.1	1190	9.4	3123	7.8
≥3	841	1.5	61	6.3	126	2.9	218	1.7	436	1.1
Townsend deprivation index										
1 (least deprived)	11,719	20.2	146	15.0	752	17.4	2445	19.4	8376	20.9
2	11,797	20.4	173	17.8	811	18.7	2515	19.9	8298	20.7
3	11,672	20.1	214	22.0	884	20.4	2586	20.5	7988	20.0
4	11,548	19.9	223	22.9	958	22.1	2486	19.7	7881	19.7
5 (most deprived)	8203	14.2	155	15.9	682	15.8	1875	14.9	5491	13.7
Unknown	3007	5.2	61	6.3	239	5.5	707	5.6	2000	5.0
Practice location										
Urban	38,467	66.4	628	64.6	2862	66.2	8,443	66.9	26,534	66.3
Town	6335	10.9	114	11.7	476	11.0	1,207	9.6	4,538	11.3
Rural	3373	5.8	55	5.7	239	5.5	703	5.6	2,376	5.9
Unknown	9771	16.9	175	18.0	749	17.3	2,261	17.9	6,586	16.5

BMI, body mass index; eGFR, estimated glomerular filtration rate; HbA_1c_, glycated hemoglobin; PCP, primary care physician.

Among the comorbidities we assessed (Table 2), hypertension was the most prevalent; over 55% of patients had hypertension. The proportion was highest among individuals with an eGFR of 15–29 mL/min (68.3%). A history of MI or IS/TIA was recorded in 9.6% and 9.8% of patients, respectively. Other frequent comorbidities included cancer (9.1%), hyperlipidemia (6.9%), heart failure (6.9%), peripheral artery disease (6.4%), atrial fibrillation (6.0%) and deep vein thrombosis (5.9%). The prevalence of comorbidities tended to be higher in patients with lower eGFRs; about a third of those with an eGFR of 15–29 mL/min (CKD stage 4) had hyperlipidemia.

**Table 2:** **Comorbidities recorded any time before the start date, overall and according to eGFR category**

			**eGFR category (mL/min)**							
	**Overall (** ***N*** **= 57,946)**		**15–29 (n = 972)**		**30–44 (n = 4326)**		**45–59 (n = 12,614)**		**≥60 (n = 40,034)**	
	**Number**	**%**	**Number**	**%**	**Number**	**%**	**Number**	**%**	**Number**	**%**
MI	5581	9.6	196	20.2	729	16.9	1499	11.9	3157	7.9
IS/TIA	5675	9.8	207	21.3	774	17.9	1677	13.3	3017	7.5
COPD	2651	4.6	85	8.7	279	6.4	725	5.7	1562	3.9
Thyroid disease	4932	8.5	145	14.9	595	13.8	1441	11.4	2751	6.9
Hypertension	32,752	56.5	664	68.3	2931	67.8	8197	65.0	20,960	52.4
Renal hypertension	29	0.1	4	0.4	7	0.2	6	0.0	12	0.0
Hyperlipidemia	3998	6.9	323	33.2	891	20.6	1342	10.6	1442	3.6
DVT	3429	5.9	106	10.9	373	8.6	933	7.4	2017	5.0
PAD	3695	6.4	158	16.3	554	12.8	1026	8.1	1957	4.9
Anemia	3561	6.1	178	18.3	539	12.5	949	7.5	1895	4.7
Atrial fibrillation	3494	6.0	67	6.9	265	6.1	742	5.9	2420	6.0
Heart failure	4011	6.9	73	7.5	313	7.2	853	6.8	2772	6.9
Peptic ulcer disease	3676	6.3	89	9.2	354	8.2	880	7.0	2353	5.9
Chronic liver disease	685	1.2	10	1.0	47	1.1	134	1.1	494	1.2
Gout	3599	6.2	134	13.8	480	11.1	876	6.9	2109	5.3
Osteoporosis	1253	2.2	37	3.8	148	3.4	386	3.1	682	1.7
Cancer	5295	9.1	119	12.2	620	14.3	1434	11.4	3122	7.8
Anxiety	6171	10.6	122	12.6	453	10.5	1354	10.7	4242	10.6
GERD	6152	10.6	121	12.4	546	12.6	1493	11.8	3992	10.0

COPD, chronic obstructive pulmonary disease; DVT, deep vein thrombosis; eGFR, estimated glomerular filtration rate; GERD, gastroesophageal reflux disease; IS, ischemic stroke; MI, myocardial infarction; PAD, peripheral arterial disease; TIA, transient ischemic attack.

### Incidences of death, myocardial infarction and stroke

**Figure 1:**
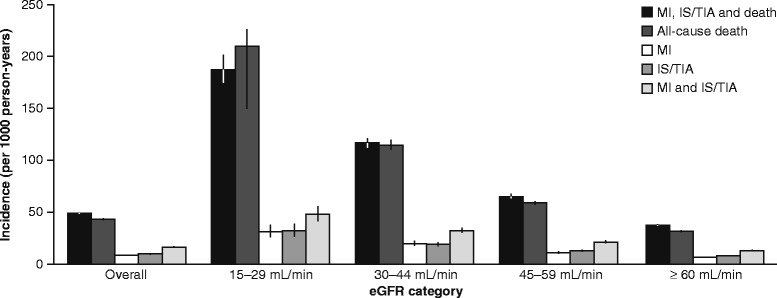
Incidence rates of death, myocardial infarction (MI) and ischemic stroke (IS)/transient ischemic attack (TIA). Incidence rates are shown both overall and according to estimated glomerular filtration rate (eGFR) category. Black vertical lines represent 95% confidence intervals.

Incidence rates of death, MI, IS/TIA and combined outcomes stratified by eGFR category and overall are shown in Figure 1.

### Mortality

**Figure 2:**
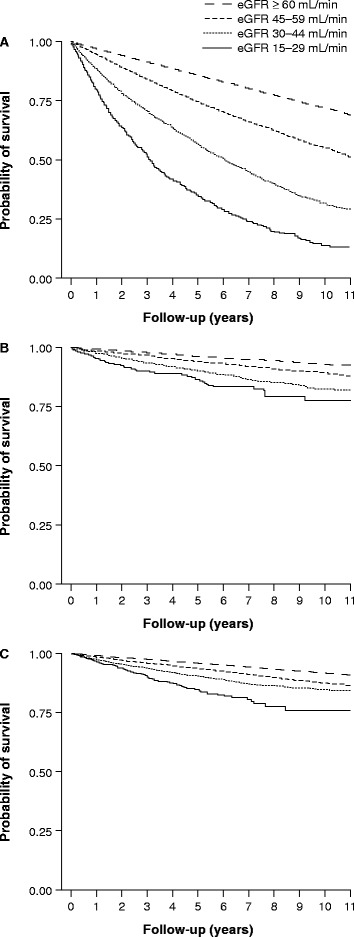
Kaplan–Meier survival estimates. Cumulative incidence of **(A)** death, **(B)** myocardial infarction and **(C)** ischemic stroke or transient ischemic attack according to estimated glomerular filtration rate (eGFR) category.

A total of 16,578 (28.6%) patients died during the study period. The person-time contribution was 379,833 person-years over a median follow-up time of 6.76 years. The overall mortality was 43.65 deaths per 1000 person-years (95% CI: 42.99–44.31). There was a marked increase in all-cause mortality with decreasing values of eGFR. Patients with an eGFR of 15–29 mL/min (CDK stage 4) showed the highest mortality (210.01 deaths per 1000 person-years [95% CI: 149.91–226.28]), whereas those with an eGFR ≥ 60 mL/min showed the lowest mortality (31.99 deaths per 1000 person-years [95% CI: 31.33–32.66]). Kaplan–Meier curves of cumulative incidence of death are shown in Figure 2A.

### Incidence of myocardial infarction

The overall incidence rate of MI was 9.26 cases per 1000 person-years (95% CI: 8.96–9.58) over a median follow-up time of 6.64 years. As for mortality, the incidence rate of MI increased with decreasing values of eGFR. The incidence rates of MI for patients with an eGFR of 15–29 mL/min (CKD stage 4) and ≥ 60 mL/min were 31.65 (95% CI: 26.02–38.51) and 7.44 (95% CI: 7.12–7.77) cases per 1000 person-years, respectively. Kaplan–Meier curves of cumulative incidence of MI are shown in Figure 2B.

### Incidence of ischemic stroke and transient ischemic attack

The overall incidence rate of IS/TIA was 10.39 cases per 1000 person-years (95% CI: 10.07–10.73) with a median follow-up time of 6.56 years and a person-time contribution of 364,258 person-years. An increased incidence rate of IS/TIA was observed with declining renal function. The incidence rates of IS/TIA were 32.48 cases per 1000 person-years (95% CI: 26.70–39.51) in patients with CKD stage 4 (eGFRs of 15–29 mL/min) and 8.65 cases per 1000 person-years (95% CI: 8.30–9.00) in patients with an eGFR ≥ 60 mL/min. Kaplan–Meier curves of cumulative incidence of IS/TIA are shown in Figure 2C.

### Cox regression analyses

Risks of death, MI and IS/TIA increased significantly with decreasing values of eGFR (Table 3). For patients with eGFR 15–29 mL/min (CKD stage 4), the adjusted HRs relative to patients with an eGFR ≥ 60 mL/min were 2.79 (95% CI: 2.57–3.03) for death, 2.33 (95% CI: 1.89–2.87) for MI and 1.77 (95% CI: 1.43–2.18) for IS/TIA. Corresponding estimates for patients with eGFRs of 45–59 mL/min were 1.25 (95% CI: 1.20–1.30), 1.27 (95% CI: 1.17–1.38) and 1.09 (95% CI: 1.01–1.18).

**Table 3:** **HRs of death, MI and IS/TIA associated with eGFR category**

	**Death**	**MI**	**IS/TIA**
	**HR** ^**a**^ **(95% CI)**	**HR** ^**a**^ **(95% CI)**	**HR** ^**a**^ **(95% CI)**
eGFR calculated with the MDRD study equation, mL/min			
15–29	2.79 (2.57–3.03)	2.34 (1.90–2.88)	1.78 (1.44–2.19)
30–59	1.38 (1.33-1.43)	1.37 (1.27-1.48)	1.13 (1.05-1.22)
30–44	1.83 (1.74–1.92)	1.75 (1.56–1.97)	1.27 (1.13–1.43)
45–59	1.25 (1.20–1.30)	1.27 (1.17–1.38)	1.09 (1.01–1.18)
≥60	1 (−)	1 (−)	1 (−)
eGFR calculated with the CKD-EPI equation, mL/min			
15–29	2.79 (2.59–2.99)	2.21 (1.83–2.66)	1.73 (1.44–2.09)
30–59	1.41 (1.36-1.46)	1.38 (1.28-1.49)	1.20 (1.12-1.29)
30–44	1.75 (1.67–1.84)	1.72 (1.54–1.92)	1.28 (1.15–1.43)
45–59	1.29 (1.24–1.34)	1.27 (1.17–1.38)	1.19 (1.09–1.27)
≥60	1 (−)	1 (−)	1 (−)

^a^Adjusted for sex, age at start date, duration of diabetes, BMI, smoking status, number of medications, HbA_1c_ level, presence of hypertension hyperlidemia, and history of MI, IS/TIA and IHD.

BMI, body mass index; CI, confidence interval; eGFR, estimated glomerular filtration rate; HbA_1c_, glycated hemoglobin; HR, hazard ratio; IHD, ischemic heart disease; IS, ischemic stroke; MI, myocardial infarction; TIA, transient ischemic attack.

HRs for death, MI and IS/TIA associated with other potential risk factors are shown in Tables 4, 5 and 6. Overall, women had a lower risk of death and of MI than men (HR: 0.80 [95% CI: 0.77–0.82] and HR: 0.71 [95% CI: 0.66–0.77], respectively) and the risk of IS/TIA was similar for men and women. For each outcome, a longer duration of diabetes was generally associated with a greater risk. Overall, the HRs associated with diabetes diagnosed more than 15 years before the start date relative to diabetes diagnosed less than 5 years before the start date were 1.50 (95% CI: 1.43–1.57) for death, 1.54 (95% CI: 1.39–1.71) for MI and 1.27 (95% CI: 1.15–1.41) for IS/TIA. Age was a strong predictor of death, MI and IS/TIA. The HRs for patients aged 75 years or older relative to patients aged 20–49 years were 11.24 (95% CI: 9.97–12.67), 2.99 (95% CI: 2.49–3.59) and 5.33 (95% CI: 4.35–6.54) for death, MI and IS/TIA, respectively. BMI did not affect the risk of MI or IS/TIA significantly. The risk of death, however, was significantly lower for overweight patients (BMI of 25–29 kg/m^2^) and obese patients (BMI ≥ 30 kg/m^2^) than for individuals with a BMI of 20–24 kg/m^2^ (HR: 0.78 [95% CI: 0.75–0.82] and HR 0.82 [95% CI: 0.78–0.85], respectively). Conversely, underweight patients (BMI of 15–19 kg/m^2^) were at higher risk of death than individuals with a BMI of 20–24 kg/m^2^ (HR: 1.51 [95% CI: 1.36–1.66]).

**Table 4:** **HRs of death associated with potential risk factors, overall and stratified by eGFR category**

	**Non-death**	**Death**		**eGFR category**			
	**n = 41,368**	**n = 16,578**	**Overall**	**15–29 mL/min**	**30–44 mL/min**	**45–59 mL/min**	**≥60 mL/min**
	**Number (%)**	**Number (%)**	**HR** ^**a**^ **(95% CI)**	**HR** ^**b**^ **(95% CI)**	**HR** ^**b**^ **(95% CI)**	**HR** ^**b**^ **(95% CI)**	**HR** ^**b**^ **(95% CI)**
Sex							
Men	22,752 (55.0)	9365 (56.5)	1 (−)	1 (−)	1 (−)	1 (−)	1 (−)
Women	18,616 (45.0)	7213 (43.5)	0.80 (0.77–0.82)	1.03 (0.87–1.21)	0.77 (0.71–0.84)	0.79 (0.75–0.84)	0.78 (0.74–0.82)
Age at start date (years)							
20–49	5428 (13.1)	295 (1.8)	1 (−)	1 (−)	1 (−)	1 (−)	1 (−)
50–74	29,403 (71.1)	8669 (52.3)	3.90 (3.47–4.39)	0.60 (0.25–1.48)	2.62 (1.24–5.54)	2.43 (1.62–3.63)	3.75 (3.30–4.25)
≥75	6537 (15.8)	7614 (45.9)	11.24 (9.97–12.67)	0.97 (0.40–2.40)	5.12 (2.43–10.80)	6.40 (4.27–9.58)	13.01 (11.42–14.83)
Duration of diabetes (years)							
<5	20,135 (48.7)	5914 (35.7)	1 (−)	1 (−)	1 (−)	1 (−)	1 (−)
5–9	12,134 (26.3)	4989 (30.1)	1.16 (1.12–1.21)	1.07 (0.87–1.32)	1.14 (1.03–1.27)	1.14 (1.06–1.22)	1.17 (1.11–1.23)
10–14	5497 (13.3)	3006 (18.1)	1.32 (1.26–1.38)	1.12 (0.88–1.42)	1.26 (1.12–1.41)	1.26 (1.16–1.37)	1.35 (1.27–1.44)
≥15	3602 (8.7)	2669 (16.1)	1.50 (1.43–1.57)	1.30 (1.04–1.62)	1.38 (1.22–1.55)	1.46 (1.34–1.60)	1.53 (1.43–1.64)
BMI (kg/m^2^)							
15–19	477 (1.2)	438 (2.6)	1.51 (1.36–1.66)	1.12 (0.63–2.00)	1.30 (0.98–1.73)	1.53 (1.27–1.84)	1.56 (1.37–1.79)
20–24	6127 (14.8)	3419 (20.6)	1 (−)	1 (−)	1 (−)	1 (−)	1 (−)
25–29	15,249 (36.9)	5762 (34.8)	0.78 (0.75–0.82)	0.72 (0.57–0.90)	0.82 (0.73–0.92)	0.79 (0.73–0.86)	0.78 (0.73–0.83)
≥30	17,577 (42.5)	5382 (32.5)	0.82 (0.78–0.85)	0.64 (0.51–0.81)	0.80 (0.71–0.90)	0.85 (0.78–0.92)	0.82 (0.77–0.87)
Unknown	1938 (4.7)	1577 (9.5)	1.43 (1.34–1.52)	1.17 (0.88–1.55)	1.32 (1.13–1.54)	1.54 (1.37–1.72)	1.42 (1.30–1.56)
Smoking status							
Non–smoker	21,930 (53.0)	8245 (49.7)	1 (−)	1 (−)	1 (−)	1 (−)	1 (−)
Current	6819 (16.5)	3309 (20.0)	1.50 (1.44–1.57)	1.09 (0.84–1.40)	1.33 (1.18–1.50)	1.60 (1.48–1.74)	1.54 (1.46–1.63)
Former	10,815 (26.1)	4196 (25.3)	1.07 (1.03–1.12)	1.04 (0.86–1.25)	1.00 (0.91–1.11)	1.09 (1.02–1.17)	1.08 (1.03–1.14)
Unknown	1804 (4.4)	828 (5.0)	0.95 (0.88–1.02)	1.06 (0.78–1.45)	0.88 (0.73–1.06)	1.00 (0.86–1.15)	0.91 (0.82–1.01)
Number of medications							
0–1	11,963 (28.9)	3044 (18.4)	1 (−)	1 (−)	1 (−)	1 (−)	1 (−)
2–4	15,169 (36.7)	5289 (31.9)	1.21 (1.15–1.26)	0.90 (0.67–1.20)	1.07 (0.94–1.23)	1.19 (1.09–1.30)	1.22 (1.16–1.30)
5–9	12,235 (29.6)	6445 (38.9)	1.45 (1.39–1.52)	1.02 (0.79–1.32)	1.15 (1.01–1.31)	1.39 (1.27–1.51)	1.53 (1.44–1.63)
≥10	2001 (4.8)	1800 (10.9)	2.03 (1.91–2.16)	1.03 (0.76–1.39)	1.67 (1.43–1.96)	2.01 (1.79–2.26)	2.18 (1.99–2.38)
HbA_1c_ (%)							
<7.00	14,151 (34.2)	5231 (31.6)	1 (−)	1 (−)	1 (−)	1 (−)	1 (−)
7.00–7.99	9363 (22.6)	3460 (20.9)	1.01 (0.97–1.05)	1.29 (1.03–1.61)	1.04 (0.92–1.16)	0.98 (0.91–1.06)	1.01 (0.95–1.07)
8.00–8.99	5333 (12.9)	1888 (11.4)	0.97 (0.92–1.02)	1.02 (0.77–1.36)	1.12 (0.97–1.29)	0.96 (0.87–1.07)	0.95 (0.88–1.02)
9.00–9.99	3319 (8.0)	1315 (7.9)	1.14 (1.07–1.21)	1.40 (1.01–1.92)	1.24 (1.05–1.46)	1.18 (1.04–1.32)	1.11 (1.02–1.20)
≥10.00	3363 (8.1)	1435 (8.7)	1.35 (1.27–1.43)	1.23 (0.92–1.65)	1.46 (1.23–1.73)	1.43 (1.27–1.60)	1.31 (1.21–1.42)
Missing	5839 (14.1)	3249 (19.6)	1.24 (1.19–1.30)	1.35 (1.09–1.67)	1.38 (1.23–1.54)	1.19 (1.09–1.30)	1.23 (1.16–1.31)
Comorbidities^c^							
Hypertension	23,223 (56.1)	9529 (57.5)	0.87 (0.84–0.90)	0.68 (0.58–0.81)	0.76 (0.70–0.83)	0.85 (0.80–0.90)	0.91 (0.87–0.95)
Hyperlipidemia	1343 (3.2)	2655 (16.0)	2.04 (1.95–2.13)	1.68 (1.42–1.98)	1.76 (1.59–1.93)	2.13 (1.97–2.31)	2.33 (2.16–2.51)
History of MI	3033 (7.3)	2548 (15.4)	1.14 (1.09–1.19)	1.14 (0.94–1.39)	1.14 (1.02–1.27)	1.09 (1.00–1.19)	1.20 (1.12–1.28)
History of IS/TIA	2776 (6.7)	2899 (17.5)	1.51 (1.45–1.57)	1.12 (0.93–1.36)	1.39 (1.26–1.54)	1.46 (1.36–1.57)	1.67 (1.57–1.77)
History of IHD^d^	6146 (14.9)	4052 (24.4)	1.02 (0.98–1.06)	1.10 (0.93–1.30)	0.92 (0.84–1.02)	1.01 (0.94–1.08)	1.03 (0.97–1.09)
COPD	1075 (2.6)	1576 (9.5)	1.77 (1.68–1.87)	1.55 (1.20–2.01)	1.32 (1.14–1.54)	1.65 (1.49–1.83)	1.95 (1.81–2.10)
Thyroid disease	3418 (8.3)	1514 (9.1)	0.92 (0.87–0.97)	0.97 (0.77–1.21)	0.89 (0.79–1.01)	0.98 (0.90–1.08)	0.90 (0.83–0.98)
DVT	2138 (5.2)	1291 (7.8)	1.12 (1.06–1.19)	0.97 (0.76–1.23)	1.10 (0.96–1.27)	1.04 (0.94–1.15)	1.17 (1.08–1.27)
PAD	1742 (4.2)	1953 (11.8)	1.35 (1.28–1.42)	1.01 (0.82–1.24)	1.19 (1.06–1.33)	1.41 (1.29–1.55)	1.44 (1.34–1.55)
Anemia	2097 (5.1)	1464 (8.8)	1.24 (1.17–1.31)	1.05 (0.86–1.28)	1.26 (1.12–1.41)	1.23 (1.11–1.36)	1.29 (1.18–1.40)
Atrial fibrillation	2483 (6.0)	1011 (6.1)	1.01 (0.95–1.07)	1.04 (0.76–1.41)	0.98 (0.83–1.16)	1.05 (0.93–1.18)	1.00 (0.91–1.09)
Heart failure	2850 (6.9)	1161 (7.0)	1.02 (0.96–1.08)	1.07 (0.79–1.44)	1.03 (0.88–1.20)	1.04 (0.93–1.17)	0.99 (0.91–1.08)
Gout	2370 (5.7)	1229 (7.4)	0.98 (0.92–1.04)	0.95 (0.76–1.19)	0.98 (0.86–1.12)	0.99 (0.88–1.10)	1.00 (0.92–1.10)
Cancer	2948 (7.1)	2347 (14.2)	1.49 (1.42–1.55)	1.50 (1.20–1.88)	1.19 (1.07–1.34)	1.34 (1.23–1.45)	1.67 (1.57–1.78)

^a^Adjusted for sex, age at start date, duration of diabetes, BMI, smoking status, number of medications, HbA1c level, presence of hypertension hyperlidemia, and history of MI, IS/TIA, IHD. and eGFR category. ^b^Adjusted for sex, age at start date, duration of diabetes, BMI, smoking status, number of medications, HbA1c level, presence of hypertension hyperlidemia, and history of MI, IS/TIA and IHD. ^c^Relative to absence of comorbidity. ^d^Excluding MI.

BMI, body mass index; CI, confidence interval; COPD, chronic obstructive pulmonary disease; DVT, deep vein thrombosis; eGFR, estimated glomerular filtration rate; HbA_1c_, glycated hemoglobin; HR, hazard ratio; IHD, ischemic heart disease; IS, ischemic stroke; MI, myocardial infarction; PAD, peripheral artery disease; TIA, transient ischemic attack.

**Table 5:** **HRs of MI associated with potential risk factors, overall and stratified by eGFR category**

	**Non–MI**	**MI**		**eGFR category**			
	**n = 54,511**	**n = 3435**	**Overall**	**15–29 mL/min**	**30–44 mL/min**	**45–59 mL/min**	**≥60 mL/min**
	**Number (%)**	**Number (%)**	**HR** ^**a**^ **(95% CI)**	**HR** ^**b**^ **(95% CI)**	**HR** ^**b**^ **(95% CI)**	**HR** ^**b**^ **(95% CI)**	**HR** ^**b**^ **(95% CI)**
Sex							
Men	29,966 (55.0)	2151 (62.6)	1 (−)	1 (−)	1 (−)	1 (−)	1 (−)
Women	25,545 (45.0)	1284 (37.4)	0.71 (0.66–0.77)	1.15 (0.74–1.78)	0.90 (0.73–1.11)	0.78 (0.68–0.90)	0.62 (0.56–0.69)
Age at start date (years)							
20–49	5573 (10.2)	150 (4.4)	1 (−)	1 (−)	1 (−)	1 (−)	1 (−)
50–74	35,880 (65.8)	2192 (63.8)	1.73 (1.46–2.05)	0.39 (0.05–2.96)	1.27 (0.31–5.17)	1.04 (0.57–1.91)	1.75 (1.46–2.10)
≥75	13,058 (24.0)	1093 (31.8)	2.99 (2.49–3.59)	0.44 (0.06–3.35)	1.77 (0.44–7.22)	1.76 (0.96–3.23)	3.26 (2.66–4.00)
Duration of diabetes (years)							
<5	24,828 (45.6)	1221 (35.6)	1 (−)	1 (−)	1 (−)	1 (−)	1 (−)
5–9	16,112 (29.6)	1011 (29.4)	1.14 (1.05–1.24)	0.55 (0.29–1.03)	1.04 (0.81–1.34)	1.20 (1.01–1.42)	1.15 (1.04–1.28)
10–14	7845 (14.4)	658 (19.2)	1.43 (1.30–1.58)	1.41 (0.79–2.52)	0.98 (0.73–1.31)	1.62 (1.34–1.95)	1.44 (1.27–1.63)
≥15	5726 (10.5)	545 (15.9)	1.54 (1.39–1.71)	1.41 (0.81–2.46)	1.44 (1.09–1.89)	1.69 (1.38–2.06)	1.47 (1.28–1.70)
BMI (kg/m^2^)							
15–19	863 (1.6)	52 (1.5)	1.25 (0.94–1.66)	–	0.73 (0.27–2.01)	1.12 (0.65–1.94)	1.50 (1.05–2.13)
20–24	8999 (16.5)	547 (15.9)	1 (−)	1 (−)	1 (−)	1 (−)	1 (−)
25–29	19,633 (36.0)	1378 (40.1)	1.08 (0.98–1.19)	1.12 (0.64–1.98)	1.20 (0.91–1.59)	1.05 (0.87–1.27)	1.06 (0.93–1.21)
≥30	21,713 (39.8)	1246 (36.3)	1.01 (0.91–1.12)	0.67 (0.37–1.22)	0.90 (0.67–1.22)	0.99 (0.81–1.22)	1.05 (0.91–1.20)
Unknown	3303 (6.1)	212 (6.2)	1.22 (1.04–1.44)	0.45 (0.16–1.24)	0.95 (0.60–1.48)	1.38 (1.02–1.87)	1.27 (1.01–1.59)
Smoking status							
Non–smoker	28,484 (52.3)	1691 (49.2)	1 (−)	1 (−)	1 (−)	1 (−)	1 (−)
Current	9427 (17.3)	701 (20.4)	1.40 (1.28–1.53)	0.84 (0.42–1.70)	1.23 (0.91–1.66)	1.41 (1.17–1.70)	1.43 (1.27–1.59)
Former	14,116 (25.9)	895 (26.1)	0.99 (0.91–1.08)	0.85 (0.52–1.39)	0.87 (0.68–1.11)	1.03 (0.88–1.21)	1.01 (0.91–1.13)
Unknown	2484 (4.6)	148 (4.3)	0.98 (0.82–1.17)	0.74 (0.26–2.14)	1.22 (0.78–1.91)	1.12 (0.80–1.56)	0.89 (0.70–1.12)
Number of medications							
0–1	14,314 (26.3)	693 (20.2)	1 (−)	1 (−)	1 (−)	1 (−)	1 (−)
2–4	19,322 (35.4)	1136 (33.1)	1.15 (1.05–1.27)	0.66 (0.31–1.40)	1.44 (1.02–2.04)	1.31 (1.08–1.59)	1.08 (0.96–1.21)
5–9	17,413 (31.9)	1267 (36.9)	1.18 (1.07–1.30)	0.96 (0.51–1.82)	1.29 (0.93–1.80)	1.22 (1.01–1.48)	1.12 (0.99–1.27)
≥10	3462 (6.4)	339 (9.9)	1.41 (1.23–1.62)	0.65 (0.30–1.44)	1.54 (1.04–2.29)	1.28 (0.97–1.69)	1.57 (1.31–1.89)
HbA_1c_ (%)							
<7.00	18,429 (33.8)	953 (27.7)	1 (−)	1 (−)	1 (−)	1 (−)	1 (−)
7.00–7.99	12,074 (22.2)	749 (21.8)	1.15 (1.05–1.27)	0.98 (0.54–1.78)	1.17 (0.90–1.52)	0.90 (0.74–1.09)	1.30 (1.14–1.48)
8.00–8.99	6721 (12.3)	500 (14.6)	1.33 (1.19–1.49)	0.83 (0.40–1.70)	1.23 (0.89–1.71)	1.22 (0.99–1.51)	1.45 (1.26–1.68)
9.00–9.99	4310 (7.9)	324 (9.4)	1.40 (1.23–1.59)	1.23 (0.54–2.76)	1.28 (0.88–1.89)	1.24 (0.96–1.60)	1.53 (1.30–1.81)
≥10.00	4468 (8.6)	330 (9.6)	1.53 (1.35–1.74)	0.82 (0.38–1.78)	0.89 (0.55–1.43)	1.38 (1.07–1.80)	1.77 (1.51–2.08)
Missing	8509 (15.6)	579 (16.9)	1.24 (1.11–1.37)	1.32 (0.76–2.30)	0.90 (0.67–1.22)	1.23 (1.01–1.50)	1.34 (1.16–1.54)
Comorbidities^c^							
Hypertension	30,684 (56.3)	2068 (60.2)	1.05 (0.98–1.13)	0.73 (0.46–1.14)	0.82 (0.67–1.01)	1.08 (0.94–1.25)	1.09 (1.00–1.20)
Hyperlipidemia	3574 (6.6)	424 (12.3)	1.39 (1.25–1.56)	1.57 (1.02–2.41)	1.53 (1.21–1.94)	1.45 (1.20–1.76)	1.31 (1.09–1.58)
History of MI	4778 (8.8)	803 (23.4)	1.94 (1.77–2.12)	2.25 (1.44–3.52)	1.80 (1.41–2.30)	1.99 (1.68–2.37)	1.93 (1.71–2.19)
History of IS/TIA	5187 (9.5)	488 (14.2)	1.29 (1.17–1.43)	1.17 (0.71–1.92)	1.19 (0.92–1.53)	1.21 (1.01–1.45)	1.42 (1.24–1.63)
History of IHD^d^	9021 (16.5)	1177 (34.3)	1.66 (1.53–1.80)	1.35 (0.88–2.08)	1.34 (1.07–1.68)	1.57 (1.35–1.83)	1.81 (1.62–2.02)
COPD	2453 (4.5)	198 (5.8)	1.17 (1.01–1.35)	0.83 (0.35–1.96)	1.13 (0.78–1.66)	1.08 (0.81–1.43)	1.24 (1.01–1.52)
Thyroid disease	4648 (8.5)	284 (8.3)	0.96 (0.85–1.09)	1.37 (0.80–2.36)	0.86 (0.64–1.17)	0.91 (0.73–1.13)	1.01 (0.84–1.21)
DVT	3199 (5.9)	230 (6.7)	1.03 (0.90–1.18)	0.43 (0.19–0.98)	1.03 (0.73–1.46)	0.88 (0.68–1.15)	1.17 (0.97–1.40)
PAD	3253 (6.0)	442 (12.9)	1.53 (1.38–1.70)	1.15 (0.70–1.91)	1.55 (1.20–2.00)	1.40 (1.14–1.71)	1.67 (1.45–1.93)
Anemia	3310 (6.1)	251 (7.3)	1.12 (0.98–1.28)	1.04 (0.63–1.73)	0.93 (0.68–1.27)	1.18 (0.94–1.50)	1.18 (0.97–1.44)
Atrial fibrillation	3273 (6.0)	221 (6.4)	1.08 (0.95–1.24)	0.96 (0.41–2.24)	0.77 (0.49–1.22)	1.07 (0.81–1.41)	1.17 (0.99–1.39)
Heart failure	3759 (6.9)	252 (7.3)	1.06 (0.93–1.21)	0.83 (0.39–1.79)	0.59 (0.37–0.95)	1.02 (0.79–1.32)	1.21 (1.03–1.42)
Gout	3359 (6.2)	240 (7.0)	0.93 (0.81–1.06)	1.25 (0.72–2.20)	0.81 (0.57–1.13)	1.17 (0.92–1.49)	0.83 (0.68–1.01)
Cancer	4986 (9.1)	309 (9.0)	1.00 (0.89–1.13)	1.61 (0.92–2.85)	0.83 (0.60–1.14)	1.07 (0.87–1.32)	0.97 (0.82–1.15)

^a^Adjusted for sex, age at start date, duration of diabetes, BMI, smoking status, number of medications, HbA1c level, presence of hypertension hyperlidemia, and history of MI, IS/TIA, IHD and eGFR category. ^b^Adjusted for sex, age at start date, duration of diabetes, BMI, smoking status, number of medications, HbA1c level, presence of hypertension hyperlidemia, and history of MI, IS/TIA, and IHD. ^c^Relative to absence of comorbidity. ^d^Excluding MI.

BMI, body mass index; CI, confidence interval; COPD, chronic obstructive pulmonary disease; DVT, deep vein thrombosis; eGFR, estimated glomerular filtration rate; HbA_1c_, glycated hemoglobin; HR, hazard ratio; IHD, ischemic heart disease; IS, ischemic stroke; MI, myocardial infarction; PAD, peripheral artery disease; TIA, transient ischemic attack.

**Table 6:** **HRs of IS or TIA associated with potential risk factors, overall and stratified by eGFR category**

	**Non–IS/TIA**	**IS/TIA**		**eGFR category**			
	**n = 54,161**	**n = 3785**	**Overall**	**15–29 mL/min**	**30–44 mL/min**	**45–59 mL/min**	**≥60 mL/min**
	**Number (%)**	**Number (%)**	**HR** ^**a**^ **(95% CI)**	**HR** ^**b**^ **(95% CI)**	**HR** ^**b**^ **(95% CI)**	**HR** ^**b**^ **(95% CI)**	**HR** ^**b**^ **(95% CI)**
Sex							
Men	30,055 (55.5)	2062 (54.5)	1 (−)	1 (−)	1 (−)	1 (−)	1 (−)
Women	24,106 (44.5)	1723 (45.5)	0.95 (0.89–1.02)	1.52 (0.94–2.46)	1.07 (0.85–1.33)	0.91 (0.80–1.04)	0.93 (0.86–1.02)
Age at start date (years)							
20–49	5614 (10.4)	109 (2.9)	1 (−)	–	–	1 (−)	1 (−)
50–74	35,722 (66.0)	2350 (62.1)	2.83 (2.32–3.43)	0.82 (0.53–1.25)	0.74 (0.60–0.92)	1.54 (0.80–2.99)	2.76 (2.25–3.39)
≥75	12,825 (23.7)	1326 (35.0)	5.33 (4.35–6.54)	1 (−)	1 (−)	2.69 (1.38–5.24)	5.95 (4.77–7.42)
Duration of diabetes (years)							
<5	24,591 (45.4)	1458 (38.5)	1 (−)	1 (−)	1 (−)	1 (−)	1 (−)
5–9	15,945 (29.4)	1178 (31.1)	1.12 (1.04–1.21)	0.99 (0.58–1.69)	1.10 (0.85–1.42)	1.12 (0.96–1.30)	1.12 (1.01–1.23)
10–14	7874 (14.5)	629 (16.6)	1.16 (1.06–1.28)	0.73 (0.37–1.43)	1.05 (0.78–1.42)	1.14 (0.95–1.37)	1.20 (1.06–1.36)
≥15	5751 (10.6)	520 (13.7)	1.27 (1.15–1.41)	1.26 (0.72–2.22)	1.35 (1.01–1.82)	1.15 (0.94–1.41)	1.29 (1.13–1.48)
BMI (kg/m^2^)							
15–19	851 (1.6)	64 (1.7)	1.18 (0.91–1.52)	1.26 (0.29–5.55)	1.65 (0.82–3.34)	1.44 (0.89–2.33)	0.98 (0.69–1.39)
20–24	8894 (16.4)	652 (17.2)	1 (−)	1 (−)	1 (−)	1 (−)	1 (−)
25–29	19,554 (36.1)	1457 (38.5)	1.01 (0.92–1.10)	0.71 (0.38–1.32)	1.09 (0.80–1.47)	1.13 (0.93–1.36)	0.97 (0.86–1.09)
≥30	21,625 (39.9)	1334 (35.2)	0.93 (0.84–1.02)	0.70 (0.39–1.27)	1.03 (0.75–1.40)	1.10 (0.91–1.34)	0.86 (0.76–0.98)
Unknown	3237 (6.0)	278 (7.3)	1.24 (1.07–1.43)	1.31 (0.63–2.69)	1.45 (0.96–2.18)	1.40 (1.06–1.85)	1.11 (0.91–1.36)
Smoking status							
Non–smoker	28,212 (52.1)	1963 (51.9)	1 (−)	1 (−)	1 (−)	1 (−)	1 (−)
Current	9463 (17.5)	665 (17.6)	1.19 (1.09–1.30)	0.74 (0.33–1.65)	1.20 (0.88–1.64)	1.13 (0.94–1.37)	1.24 (1.11–1.38)
Former	14,046 (25.9)	965 (25.5)	1.03 (0.95–1.11)	1.09 (0.66–1.81)	0.80 (0.62–1.04)	0.91 (0.78–1.07)	1.12 (1.01–1.24)
Unknown	2440 (4.5)	192 (5.1)	1.06 (0.91–1.24)	1.66 (0.81–3.40)	1.17 (0.75–1.83)	1.14 (0.85–1.54)	0.97 (0.79–1.20)
Number of medications							
0–1	14,249 (26.3)	758 (20.0)	1 (−)	1 (−)	1 (−)	1 (−)	1 (−)
2–4	19,205 (35.5)	1253 (33.1)	1.14 (1.04–1.25)	1.48 (0.68–3.23)	1.02 (0.72–1.46)	1.17 (0.97–1.41)	1.12 (1.00–1.25)
5–9	17,266 (31.9)	1414 (37.4)	1.28 (1.16–1.40)	1.06 (0.51–2.20)	1.11 (0.80–1.54)	1.16 (0.97–1.40)	1.34 (1.20–1.51)
≥10	3441 (6.4)	360 (9.5)	1.65 (1.44–1.89)	1.15 (0.50–2.62)	1.42 (0.96–2.09)	1.77 (1.38–2.27)	1.64 (1.36–1.97)
HbA_1c_ (%)							
<7.00	18,214 (33.6)	1168 (30.9)	1 (−)	1 (−)	1 (−)		1 (−)
7.00–7.99	12,029 (22.2)	794 (21.0)	1.02 (0.93–1.12)	1.56 (0.89–2.74)	0.95 (0.72–1.25)	0.96 (0.81–1.14)	1.04 (0.92–1.17)
8.00–8.99	6730 (12.4)	491 (13.0)	1.12 (1.00–1.24)	0.72 (0.31–1.63)	1.17 (0.83–1.63)	0.93 (0.75–1.15)	1.22 (1.06–1.39)
9.00–9.99	4297 (7.9)	337 (8.9)	1.30 (1.15–1.47)	1.15 (0.46–2.87)	1.24 (0.84–1.83)	1.32 (1.03–1.68)	1.33 (1.14–1.56)
≥10.00	4474 (8.3)	324 (8.6)	1.33 (1.17–1.51)	1.17 (0.53–2.58)	0.72 (0.42–1.24)	1.13 (0.86–1.48)	1.52 (1.31–1.77)
Missing	8417 (15.5)	671 (17.7)	1.20 (1.09–1.33)	1.36 (0.77–2.41)	0.96 (0.72–1.30)	1.15 (0.96–1.39)	1.28 (1.13–1.44)
Comorbidities^c^							
Hypertension	30,384 (56.1)	2368 (62.6)	1.07 (1.00–1.14)	0.81 (0.52–1.28)	1.06 (0.84–1.33)	1.09 (0.95–1.25)	1.07 (0.98–1.16)
Hyperlipidemia	3648 (6.7)	350 (9.2)	1.23 (1.09–1.39)	1.79 (1.15–2.79)	1.42 (1.10–1.82)	1.21 (0.98–1.48)	1.17 (0.96–1.43)
History of MI	5134 (9.5)	447 (11.8)	0.97 (0.87–1.08)	1.12 (0.65–1.91)	0.77 (0.57–1.03)	1.06 (0.87–1.30)	0.98 (0.84–1.14)
History of IS/TIA	4710 (8.7)	965 (25.5)	3.26 (3.02–3.52)	1.97 (1.25–3.12)	2.89 (2.33–3.59)	3.09 (2.68–3.57)	3.57 (3.22–3.95)
History of IHD^d^	9308 (17.2)	890 (23.5)	1.16 (1.07–1.27)	0.81 (0.50–1.31)	1.45 (1.15–1.83)	1.06 (0.91–1.24)	1.18 (1.05–1.32)
COPD	2458 (4.5)	193 (5.1)	1.04 (0.89–1.20)	1.15 (0.56–2.39)	0.79 (0.50–1.26)	1.07 (0.81–1.41)	1.03 (0.85–1.26)
Thyroid disease	4571 (8.4)	361 (9.5)	1.01 (0.90–1.13)	0.72 (0.40–1.31)	0.97 (0.72–1.29)	0.99 (0.81–1.21)	1.07 (0.91–1.25)
DVT	3139 (5.8)	290 (7.7)	1.15 (1.02–1.29)	0.67 (0.32–1.39)	1.50 (1.09–2.07)	1.30 (1.05–1.60)	1.02 (0.85–1.21)
PAD	3330 (6.1)	365 (9.6)	1.22 (1.09–1.36)	0.96 (0.54–1.68)	1.35 (1.02–1.77)	1.36 (1.11–1.67)	1.15 (0.98–1.35)
Anemia	3298 (6.1)	263 (6.9)	1.04 (0.92–1.19)	0.82 (0.47–1.44)	1.01 (0.74–1.38)	1.05 (0.83–1.33)	1.08 (0.90–1.30)
Atrial fibrillation	3273 (6.0)	221 (5.8)	0.95 (0.83–1.09)	0.61 (0.22–1.68)	0.82 (0.52–1.29)	0.85 (0.63–1.13)	1.05 (0.89–1.24)
Heart failure	3755 (6.9)	256 (6.8)	0.97 (0.86–1.11)	0.76 (0.30–1.91)	1.08 (0.74–1.57)	1.06 (0.83–1.34)	0.95 (0.80–1.12)
Gout	3299 (6.1)	300 (7.9)	1.17 (1.04–1.32)	0.86 (0.47–1.59)	1.15 (0.84–1.59)	1.34 (1.07–1.66)	1.11 (0.94–1.32)
Cancer	4845 (8.9)	450 (11.9)	1.30 (1.18–1.44)	0.84 (0.42–1.69)	1.33 (1.01–1.75)	1.09 (0.90–1.33)	1.43 (1.25–1.63)

^a^Adjusted for sex, age at start date, duration of diabetes, BMI, smoking status, number of medications, HbA1c level, presence of hypertension hyperlidemia, and history of MI, IS/TIA, IHD and eGFR category. ^b^Adjusted for sex, age at start date, duration of diabetes, BMI, smoking status, number of medications, HbA1c level, presence of hypertension hyperlidemia, and history of MI, IS/TIA, and IHD. ^c^Relative to absence of comorbidity. ^d^Excluding MI.

BMI, body mass index; CI, confidence interval; COPD, chronic obstructive pulmonary disease; DVT, deep vein thrombosis; eGFR, estimated glomerular filtration rate; HbA_1c_, glycated hemoglobin; HR, hazard ratio; IHD, ischemic heart disease; IS, ischemic stroke; MI, myocardial infarction; PAD, peripheral artery disease; TIA TIA, transient ischemic attack.

Patients with a history of MI had a greater risk of MI (HR: 1.94 [95% CI: 1.77–2.12]) than patients without such a history. Similarly, a history of IS/TIA was a strong predictor of recurrent IS/TIA (HR: 3.27 [95% CI: 3.03–3.53]). Hyperlipidemia was associated with an increased risk of death (HR: 2.03 [95% CI: 1.94–2.13]), MI (HR: 1.39 [95% CI: 1.25–1.56]) and IS/TIA (HR: 1.23 [95% CI: 1.09–1.38]), but hypertension was not. A general trend for increased risk of death, MI and IS/TIA associated with increasing HbA_1c_ levels was observed. For patients with HbA_1c_ levels ≥ 11%, the adjusted HRs relative to patients with HbA_1c_ levels < 7% were 1.43 (95% CI: 1.33–1.55) for death, 1.63 (95% CI: 1.37–1.93) for MI and 1.66 (95% CI: 1.42–1.94) for IS/TIA.

## Discussion

In a large population of patients with type 2 diabetes, incidence rates of death and cardiovascular events for each eGFR category were higher than those reported for patients with CKD in the general population [1], suggesting that diabetes adds to the burden of CKD. This may be explained in part by the higher prevalence of known risk factors for death and cardiovascular events in patients with diabetes and impaired renal function, including obesity, hypertension, hyperlipidemia and history of cardiovascular events.

In the present study, a reduced eGFR was a strong and independent risk factor for death and cardiovascular events. The association between lower eGFRs and increased all-cause mortality was consistent with observations from previous studies in various populations of patients with diabetes [1,20-26]. An association between renal impairment and increased risk of cardiovascular events was also observed in an observational study from the Swedish National Diabetes Register [25]. This study, however, excluded patients with CKD stages 4 and 5 (eGFR < 30 mL/min). Reduced eGFR was also identified as a risk factor for cardiovascular events in a small US population of patients with type 2 diabetes [26]. These observations may be explained by common features in the pathophysiologies of CKD and type 2 diabetes. Risk factors for cardiovascular events such as increased levels of procoagulant biomarkers, anemia and endothelial dysfunction have been shown to be associated with both reduced kidney function [40-42] and type 2 diabetes [43-45]. These factors may act synergistically to increase the risk of cardiovascular events compared with CKD or type 2 diabetes alone. The association of renal disease with hypoglycemia in patients with type 2 diabetes is also linked to an increased risk of cardiovascular events [46].

Our study also showed that age and duration of diabetes were predictors of all-cause mortality and incidence of cardiovascular events, irrespective of eGFR. This is in line with results from others [47] and suggests that, as the population ages and survival of patients with diabetes increases, further efforts will be required to complement ongoing measures to reduce all-cause mortality and risk of cardiovascular complications and in patients with type 2 diabetes. Traditional cardiovascular risk factors, including smoking, hyperlipidemia and a history of cardiovascular events, were also associated with an increased risk of cardiovascular events and a higher mortality in patients with type 2 diabetes. These findings echoed results from other population-based studies [48,49].

In contrast, overweight and obese people (BMI ≥ 25 kg/m^2^) had a lower mortality than individuals with a BMI of 20–24 kg/m^2^. Although counterintuitive and controversial, this ‘obesity paradox’ has been observed in several cohort studies, patient registries and clinical trial populations [50].

Overall, our results support the current UK guidelines [51], which recommend monitoring renal function annually in all individuals with type 2 diabetes, regardless of the presence or absence of nephropathy. The guidelines also recommend addressing traditional cardiovascular risk factors such as hyperlipidemia and smoking, which we found to be associated with higher risks of death, MI and IS/TIA in this population.

The present study has several strengths. THIN is a large database representative of the UK population and has been validated for use in epidemiological studies [29,30]. It has previously been used to study individuals with diabetes [3,52-54] and patients with CKD [31]. The suitability of THIN for this study is reinforced by the fact that laboratory test results are reliably and routinely recorded in the database; 90% of patients in our large and diverse cohort had a valid serum creatinine measurement. Our results from a primary care database may also be more generalizable than studies from selected populations such as referred patients, recruited cohorts or clinical trial participants. Other strengths of our study include a long follow-up period and careful ascertainment of MI and IS/TIA cases. This was deemed particularly important for IS/TIA in order to mitigate the observed tendency of Read codes to overestimate the number of IS/TIA cases. In common with all observational studies, however, ours may suffer from uncontrolled confounding. Although we tried to minimize this by adjusting results for several potential risk factors, residual confounding cannot be ruled out. It should also be noted that the levels of urine albumin were not systematically reported in THIN during the study period and it was therefore impossible to adjust analyses for this potential confounder [55,56].

NICE guidelines on the management of CKD were updated in January 2015 and now recommend the use of the CKD-EPI equation for the calculation of eGFR from serum creatinine concentration [57]. During the study period (2000–2005), however, the MDRD and the Cockcroft–Gault equations were routinely used; the MDRD equation was used in THIN and recommended by NICE and was therefore selected for the present study. Additionally, the MDRD equation has been shown to be more accurate than the Cockcroft–Gault formula in patients with CKD and diabetes [58]. The MDRD equation has also previously been used in a study of CKD in THIN [31]. It should be noted, however, that eGFR was calculated from a single serum creatinine measurement. To estimate the extent of eGFR misclassification, patients with a valid serum creatinine concentration recorded between 91 and 366 days after their start date were identified (n = 47,022, 81% of the study population). Among those patients, 14,528 had an eGFR < 60 mL/min on their start date, and the diagnosis of impaired renal function was confirmed by subsequent creatinine measurement in 12,055 individuals (83%). Conversely, 90% of patients (n = 29,240) who had an eGFR ≥ 60 mL/min on their start date and who had a valid creatinine measurement in the 91–366-day period following their start date remained in the same eGFR category. The fact that ethnicity is not recorded in THIN may also have led to misclassification; eGFR may have been underestimated in black people.

## Conclusions

In conclusion, this retrospective study based on a UK primary care database confirms the high prevalence of impaired renal function in patients with type 2 diabetes. Our findings show that all-cause mortality and the risk of cardiovascular events increase significantly with decreasing values of eGFR. In line with current UK guidelines for the treatment of type 2 diabetes, our results suggest that physicians should closely monitor renal function in patients with type 2 diabetes and initiate lifestyle changes and/or medication to delay progression of CKD and prevent end-stage renal disease. Management of associated cardiovascular risks such as hyperlipidemia and smoking should also be adequately addressed, given the very high risk of adverse cardiovascular events in patients with both type 2 diabetes and impaired renal function.

## Ethics, consent and permissions

We used The Health Improvement Network (THIN) primary care data for this study. The company that owns THIN (Cegedim Strategic Data Medical Research) has received ethical approval from the South East Research Ethics Committee (REC) to supply anonymized, pre-collected primary care data for scientific research. Patients can opt out of having their depersonalized records collected and therefore patient consent is not required when working with anonymized records in the THIN database.

## Additional file

Additional file 1:
**Ischemic stroke ascertainment.**

## Abbreviations

BMI: Body mass index
CI: Confidence interval
CKD: Chronic kidney disease
EGFR: Estimated glomerular filtration rate
HbA_1c_: Glycated hemoglobin
HR: Hazard ratio
IS/TIA: Ischemic stroke or transient ischemic attack
MDRD: Modification of diet in renal disease
MI: Myocardial infarction
MREC: Multicentre Research Ethics Committee
PCP: Primary care physician
THIN: The Health Improvement Network
