# Supplementary material for: Cardiovascular events and all-cause mortality in a cohort of 57,946 patients with type 2 diabetes: associations with renal function and cardiovascular risk factors
Source: Cardiovasc Diabetol. 2015 Apr 18;14:38. doi: 10.1186/s12933-015-0204-5 (PMC4409775; doi:10.1186/s12933-015-0204-5)
Supplement: Additional file 1: — Ischemic stroke ascertainment. [file 12933_2015_204_MOESM1_ESM.pdf]

Additional file 1

Ischemic stroke ascertainment

A computer search using Read codes suggestive of ischemic stroke or transient ischemic attack (IS/TIA) found 4799 potential cases. To validate the diagnosis of IS/TIA, we used a multistep approach (Additional Figure 1).

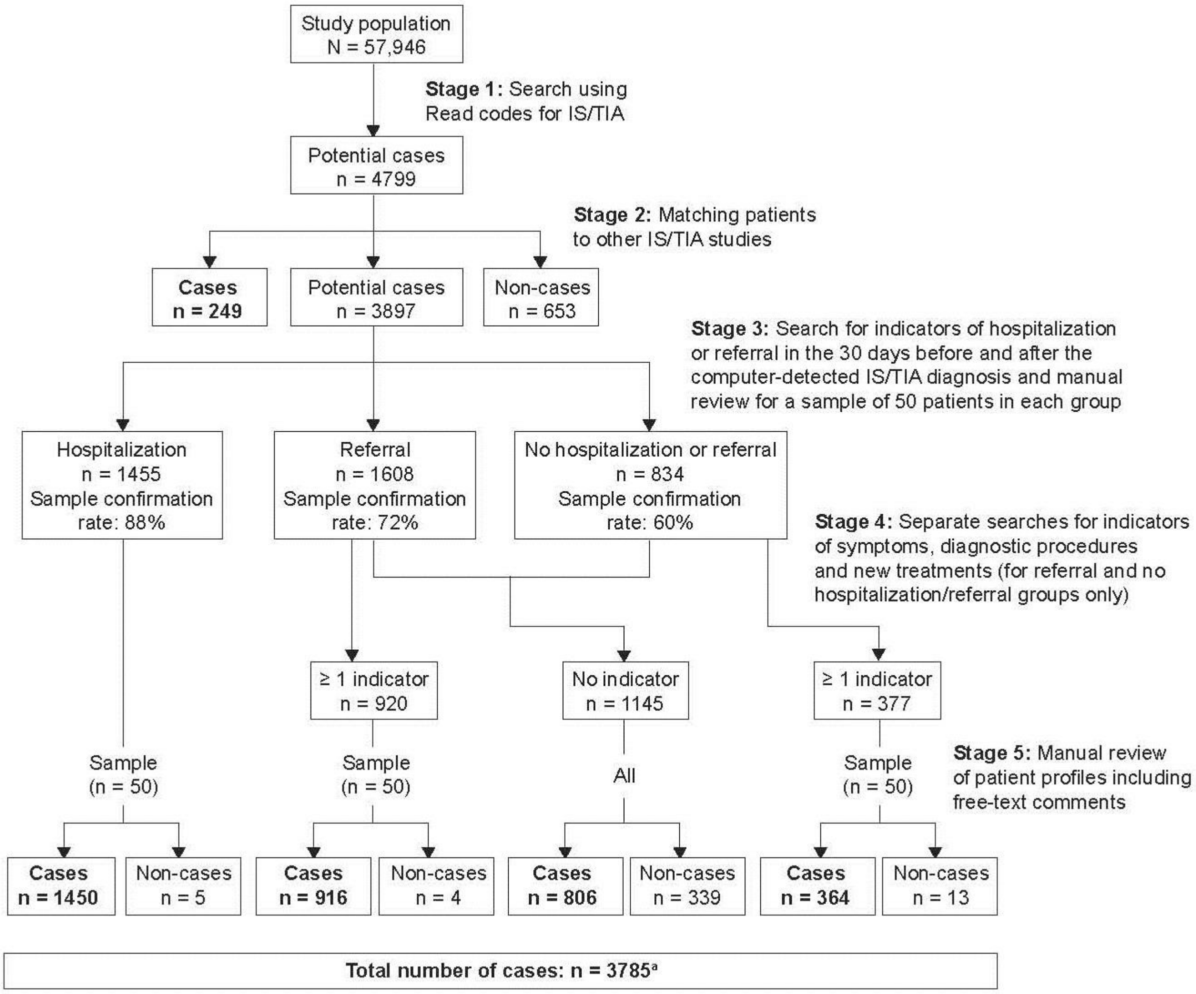

Additional Figure 1 Ascertainment of ischemic stroke (IS) and transient ischemic attack (TIA) cases. In stage 5, non-cases identified after manual review of profiles in each sample were excluded; all other patients were considered as cases. [a]Sum of numbers of cases in bold at each stage.

In the first instance, we compared our set of potential cases (n = 4799) with all patients reviewed in other projects in which we looked at a diagnosis of IS/TIA in The Health Improvement Network (THIN) [1,2]. From a total of 902 matched pairs, 653 were previously classified as non-cases and 249 as cases.

For the remaining 3897 computer-detected IS/TIA potential cases, we searched the database for any indicators of hospitalization or referral in the 30 days before and after the date of the computer-detected IS/TIA diagnosis. Patients were then classified into three mutually exclusive subgroups: the hospitalization subgroup for individuals with a code suggestive of hospitalization with or without referral (n = 1455); the referral subgroup for individuals with a code suggestive of referral without hospitalization (n = 1608); and the non-hospitalization/non-referral subgroup for the remaining individuals (n = 834). A manual review (without free-text comments) was carried out in a random sample of 50 patients in each of these three subgroups to estimate the extent of misclassification. The diagnosis of IS/TIA was considered valid if we identified any symptom related to the outcome (e.g. hemiplegia, speechlessness, being off balance, falls), any diagnostic procedure relevant to IS/TIA (e.g. computerized tomography [CT], Doppler ultrasonography), *de novo* prescription of antiplatelet and/or anticoagulant medication, or a change in antiplatelet and/or anticoagulant medication. Using these criteria, the proportions of individuals identified as probable cases from these samples were 88% (n = 44) in the hospitalization group, 72% (n = 36) in the referral group and 60% (n = 30) in the non-hospitalization/non-referral group. Owing to the high confirmation rate in the hospitalization group, all patients in this group (n = 1455) were considered as potential cases at this stage. The lower confirmation rates in the other two groups warranted further validation.

For the referral and non-hospitalization/non-referral subgroups, we identified all individuals meeting one or more of the following criteria: Read code suggestive of a diagnostic procedure relevant to IS/TIA (e.g. CT, Doppler ultrasonography or fundoscopy) in the 30 days before or after the date of the computer-detected IS/TIA diagnosis; Read code suggestive of symptoms related to a diagnosis of IS/TIA (e.g. hemiplegia, weakness, dizziness, migraine) in the 30 days before or after the date of the computer-detected IS/TIA diagnosis; or prescription of an antiplatelet agent (i.e. low-dose acetylsalicylic acid, clopidogrel and dipyridamole, separately) or warfarin from the date of the computer-detected IS/TIA diagnosis to 60 days after. In this fourth stage, we found a total of 1297 patients meeting at least one of the above criteria (920 in the referral group and 377 in the non-hospitalization/non-referral group). At this stage, these were considered to be probable cases.

Finally, we requested free-text comments for the following: a random sample of 50 patients among probable cases in the hospitalization subgroup (n = 1455); a random sample of 50 patients among probable cases in the referral subgroup (n = 920); a random sample of 50 patients among probable cases in the non-hospitalization/non-referral subgroup (n = 377); and all remaining patients not considered as probable cases after the previous stages (n = 1145). The manual review of free-text comments revealed 45 and 46 cases of IS/TIA in the samples of 50 patients from the hospitalization and referral subgroups, respectively (confirmation rate: 90% and 92%, respectively), thereby supporting our case ascertainment without free-text comments. The confirmation rate in the sample of 50 patients from the non-hospitalization/non-referral subgroup was 74%. Of all remaining patients (n = 1145), 806 (70%) were confirmed as cases; remaining non-confirmed cases were excluded from the analysis.

Overall, the final list of cases of IS/TIA comprised 3785 individuals.

References

1. Ruigomez A, Martin-Merino E, García Rodríguez LA: **Validation of ischemic cerebrovascular diagnoses in the health improvement network (THIN).** *Pharmacoepidemiol Drug Saf* 2010, **19:**579–585.
2. García Rodríguez LA, Cea Soriano L, Hill C, Johansson S: **Increased risk of stroke after discontinuation of acetylsalicylic acid: a UK primary care study.** *Neurology* 2011, **76:**740–746.
